# In vitro measurement of proton RBE: A multi-centric comparison using a harmonized setup

**DOI:** 10.1016/j.ctro.2025.100978

**Published:** 2025-05-11

**Authors:** Olga Sokol, Anders Tobias Frederiksen, Mateusz Sitarz, Brita Singers Sørensen, Elham Santina, Christopher Smith, John-William Warmenhoven, Marie Davídková, Anna Jelínek Michaelidesová, Irina Danilová, Oldřich Zahradníček, Amélia Maia Leite, Ludovic de Marzi, Frederic Pouzoulet, Paweł Olko, Justyna Miszczyk, Barbara Orzechowska, Eleftherios Papalanis, Mehran Hariri, Diana Spiegelberg, Bo Stenerlöw, Alexandru Dasu, Elisabeth Bodenstein, Elke Beyreuther, Jörg Pawelke, Dorothee Pfitzmann, Karen Kirkby, Nicholas Henthorn, Marco Durante

**Affiliations:** a GSI Helmholtz Centre for Heavy Ion Research, Darmstadt, Germany; b Department of Experimental Clinical Oncology, Aarhus University Hospital, Aarhus, Denmark; c Danish Centre for Particle Therapy (DCPT), Aarhus University Hospital, Aarhus Denmark; d CIMAP, CEA-CNRS-ENSICAEN-UNICAEN, Normandie Université, Caen, France; e Division of Cancer Sciences, Faculty of Biology, Medicine and Health, The University of Manchester, Manchester, UK; f Manchester Academic Health Science Centre, The Christie NHS Foundation Trust, Manchester, UK; g Nuclear Physics Institute of the Czech Academy of Sciences, Řež, Czech Republic; h Inserm U1021 - CNRS UMR 3347, Institut Curie, PSL Research University, University Paris Saclay, Orsay, France; i Institut Curie, PSL Research University, Radiation Oncology Department, Proton Therapy Centre, Centre universitaire, Orsay, France; j Inserm U1288, Laboratory of Translational Imaging in Oncology (LITO), Institut Curie, PSL Research University, University Paris Saclay, Orsay, France; k Translational Research Department, Institut Curie, Experimental Radiotherapy Platform, University Paris Saclay, Orsay, France; l Institute of Nuclear Physics Polish Academy of Sciences, Krakow, Poland; m Department of Immunology, Genetics and Pathology, Uppsala University, Uppsala, Sweden; n The Skandion Clinic, Uppsala, Sweden; o Helmholtz-Zentrum Dresden – Rossendorf (HZDR), Institute of Radiooncology - OncoRay, Dresden, Germany; p OncoRay – National Center for Radiation Research in Oncology, Faculty of Medicine and University Hospital Carl Gustav Carus, Technische Universität Dresden, Helmholtz-Zentrum Dresden – Rossendorf, Dresden, Germany; q Helmholtz-Zentrum Dresden – Rossendorf, Institute of Radiation Physics, Dresden, Germany; r Department of Radiotherapy and Radiation Oncology, Faculty of Medicine and University Hospital Carl Gustav Carus, Technische Universität Dresden, Dresden, Germany; s OncoRay – National Center for Radiation Research in Oncology, Faculty of Medicine, Dresden, Germany; t Institute for Condensed Matter Physics, Technische Universität Darmstadt, Darmstadt, Germany

**Keywords:** Relative biological effectiveness, Proton therapy, Cell survival

## Abstract

**Background and purpose:**

This study presents a multi-center comparison of in vitro cell survival measurements and RBE calculations following proton irradiations conducted under harmonized experimental conditions across six European institutions participating in the INSPIRE framework.

**Materials and methods:**

V79-4 cells were irradiated using spread-out Bragg peak (SOBP) proton fields of two configurations delivering 6 and 8 Gy with widths of 6 and 4 cm, respectively. Each center adhered to a standardized protocol, utilizing the same phantom design to minimize uncertainties related to sample positioning. X-ray reference irradiations were also performed to assess cell radiosensitivity across the participating centers.

**Results:**

Despite the consistent protocol, significant inter-institutional variability was observed in the survival measurements. For both treatment plans, the largest variation was detected in the most distal points of the SOBP (coefficients of variation of 43 % and 60 % for the 6 Gy and 8 Gy plans, respectively). Kruskal-Wallis statistical test confirmed the significant differences between the centers for each of the measured position in the proton field for both SOBP configurations. Discrepancies were observed in calculated RBE data as well, albeit preserving the expected trend for the values to slightly increase towards the distal edge of the SOBP (up to 1.5 and 1.3 for the 6 Gy and 8 Gy plans, respectively).

**Conclusion:**

The results of the study highlight the minimal biological variation one could expect performing proton RBE measurements in well-aligned experimental conditions and challenges in conducting large-scale, multi-center radiobiological experiments and inter-comparisons between literature data sets.

## Highlights

• This study evaluated cell survival and proton RBE using a harmonized protocol.
• Experiments were conducted across European centers with a single cell line.
• Data variability was observed despite standardized procedures and shared phantoms.
• The findings emphasize the need for cautious interpretation of literature data.

## Introduction

Proton therapy is a rapidly evolving modality in cancer treatment [1,2], offering unique dosimetric advantages over conventional photon radiotherapy due to its ability to deliver high doses to tumors while sparing surrounding healthy tissue through the Bragg peak effect. The biological response to proton irradiation, however, is still a subject of ongoing research, with the relative biological effectiveness (RBE) being a key factor. Although a fixed RBE of 1.1 is commonly applied in clinical practice [3], accumulating evidence suggests that RBE varies with proton energy, tissue type, and further biological factors, complicating treatment planning and outcome prediction [4,5]. While there are multiple models to predict the proton RBE [[6], [7], [8], [9], [10], [11]], the discrepancies in the experimental proton data [5,[12], [13], [14], [15], [16]], likely caused by variability in experimental conditions, such as beam parameters, irradiation setups, and biological sample handling, undermine the reliability of these models and hamper the implementation of the variable RBE in treatment planning.

The European project “Infrastructure in Proton International Research” (INSPIRE), with a consortium comprising 17 partners, was created to allow access to “state-of-the-art” European proton therapy research capabilities [17]. Fostering the collaboration between centers to standardize research protocols, enhance reproducibility, and enable cross-center comparisons of proton therapy data was one of the consortium goals. In this regard, one of the initiatives within INSPIRE was an intercomparison experiment designed to harmonize experimental conditions and protocols across European centers and to assess the level of the discrepancies in resulting proton RBE measurements.

In this study, we report the results of an experiment conducted by six European centers within the INSPIRE framework. The experiment focused on measuring the in vitro survival of V79-4 cells irradiated with proton beams in a spread-out Bragg peak (SOBP) configuration, with harmonized irradiation setups, cell culture protocols, and post-irradiation processing methods. The overarching aim was to evaluate the reproducibility of proton radiobiological data across multiple institutions and provide insights into the potential sources of variability in multi-center studies.

## Materials and methods

### Participating centers

Out of eight centers within the INSPIRE framework, which agreed to perform the measurements within the project timeframe (Table 1), six were able to provide a complete set of data for at least one proton field configuration. Later throughout the manuscript the data from these six centers is reported anonymously by assigning a number to each of them.

**Table 1:** List of the intercomparison experiment participants.

**Name of the institute**	**City**	**Country**
Aarhus University/ The Danish Centre for Particle Therapy	Aarhus	Denmark
University Proton Therapy Dresden / OncoRay	Dresden	Germany
Institute Curie	Orsay	France
GSI Helmholtz Centre for Heavy Ion Research	Darmstadt	Germany
Nuclear Physics Institute of the Czech Academy of Sciences	Řež	Czech Republic
Skandion Clinic / Uppsala University	Uppsala	Sweden
University of Manchester / Christie NHS Foundation Thrust	Manchester	United Kingdom
Institute of Nuclear Physics, Polish Academy of Sciences	Krakow	Poland

### Experimental setup

Each center has conducted the irradiations locally at their facility, providing the data on proton treatment plans, measured proton and reference X-ray cell survival values. The number of independent biological repetitions of the experiments performed by every center is indicated in Supplementary Table 1.

Experiments were performed using the V79-4 (Chinese hamster lung fibroblast) cell line, the widely used in vitro model in radiobiology, that was purchased by every participating center individually from the same distributor (ATCC designated as CCL-93^TM^).

**Fig. 1:**
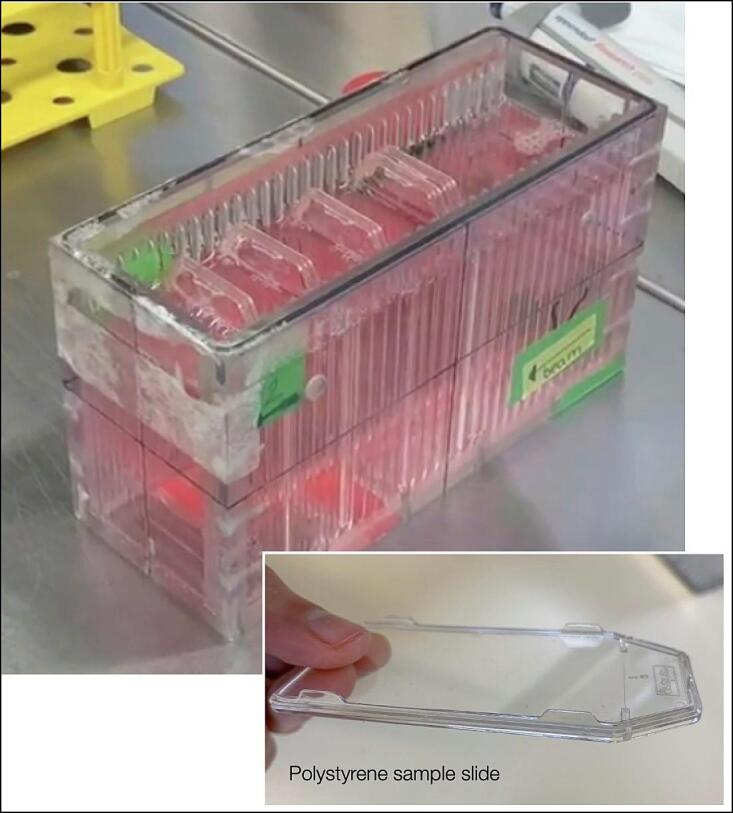

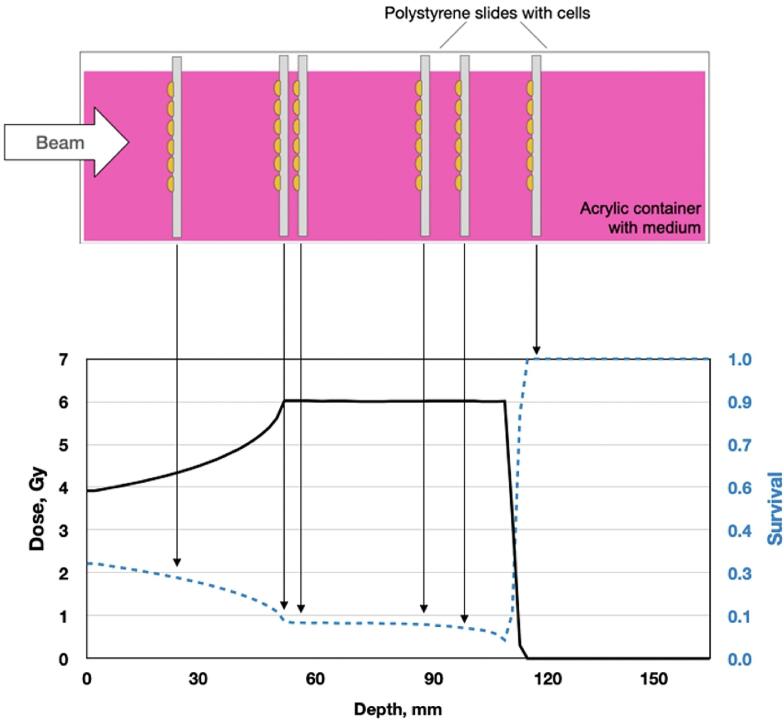
Experimental setup. (A) Acrylic phantom used for cell irradiation with proton beams. (B) Sketch of the experimental procedure for proton irradiation, where slides in the phantom were simultaneously irradiated at different positions within the SOBP.

To minimize the impact of the institute-specific irradiation setup on the proton measurements, every participant has used the same phantom for irradiations, previously developed at GSI [18] (Fig. 1A), which was circulated between the facilities. The phantom of 5 cm x 10 cm x 16 cm is made of acrylic glass and contains adjustable slots for polystyrene slides supporting the cell layers. The slot location has a resolution of 5 mm along the beam direction, while the rest of the space is filled with cell culture medium. This phantom design allowed for simultaneous survival measurements at several depth positions inside the proton field (Fig. 1B), minimizing the uncertainties associated with repositioning of samples or phantom itself. The total number of cell plates and the fields geometries were defined in the joint protocol.

All experiments were conducted on cells from passages 10–20. A total of 50,000 cells inside a 0.5 ml drop of medium were plated centrally onto the slides approximately 24 h before the irradiation. After the attachment of the cells, DMEM growth medium, supplied with 10 % fetal bovine serum and 1 % penicillin–streptomycin, was added to prevent the culture from drying. While the concentrations for the medium supplements were standardized, the centres were free to purchase them from the suppliers of their choice. Before the irradiation, cell slides were placed in the phantom as schematically shown on Fig. 1b. The number of slides per phantom was limited to eight to avoid significantly longer waiting times during the post-processing. In case of multiple irradiations following one after another (biological repetitions or different SOBP configurations), the medium in the phantom box was exchanged before insertion of the new cell samples. After the irradiation, the slides were extracted and the cells were trypsinized, followed by the counting of the cell concentration in the suspension for every sample. After that, for each dose, the corresponding number of cells were re-seeded in triplicates into 25 cm^2^ flasks pre-filled with 5 ml of medium. All the centers have used the same cell densities for re-seeding, which were calculated based on the expected survival estimated with the GSI in-house software TRiP98 [19,20]. The samples were left for 7 days incubation for the colony forming assay. Standard cutoff-point of colonies with more than 50 cells were counted.

The reference X-ray experiments were performed in the 25 cm^2^ culture flasks utilizing the same cell plating (50,000 cells centrally plated inside the 0.5 ml drop of medium 24 h before the irradiation) and post-processing procedures as in the proton experiment. The cells were trypsinized, counted inside the suspension, and re-seeded in triplicates into 25 cm^2^ flasks containing 5 ml of medium, with the cell densities for re-seeding estimated based on the published V79 survival measurements [21]. After 7 days of incubation, the surviving colonies were counted, and the survival values were fit using the linear-quadratic (LQ) model [22].

### Irradiation conditions

Table 2 summarizes the irradiation conditions for the proton and X-ray experiments conducted at each center. Reference X-ray irradiations were performed using either a 6 MV LINAC where possible (Centers 1, 2, 3 and 5), or kilovoltage X-ray generators (Centers 4 and 6) at a dose rate of approximately 2 Gy/min. For proton irradiations, centers employing active scanning (Centers 1–5) reported the range of proton energies used, along with any additional range shifters if required. In contrast, for passive scattering (Center 6), the primary proton beam energy was specified. The specifics of the quality assurance procedure for dose delivery validation were not harmonized and each center had followed their own in-house protocols.

**Table 2:** Summary of beam delivery capabilities at each participating center. WET stands for the water-equivalent thickness of the range shifter blocks or bolus introduced at several centers.

**Center**	**X-ray setup**	**Proton setup** Passive scattering (P): primary energy or Active scanning (A): energy range	
		6 Gy SOBP	8 Gy SOBP
1	6 MV Linac	A, 130.7 – 167.5 MeV, (+ 7.41 cm WET range shifter)	A, 105.5 – 137 MeV
2	6 MV Linac	A, 80.9–122.9 MeV	A, 106.9–132.1 MeV
3	6 MV Linac	A, 83.0–122.6 MeV	A, 109.5–132.6 MeV
4	200 kVp X-ray tube (3 mm Be + 3 mm Al + 0.5 mm Cu filtering)	A, 114.4–151.5 MeV (+ 4.66 cm WET range shifter)	A, 107.3–132.6 MeV
5	6 MV Linac	A, 132–173 MeV (+ 6.5 cm PMMA range shifter)	
6	250 kVp X-ray tube (7 mm Be + 1 mm Al + 1 mm Cu filtering)		P, 150 MeV (+ 32 mm WET bolus)

The experimental setup for simultaneous proton irradiations of multiple cell monolayers at different depths along the spread-out Bragg peak (SOBP) is summarized schematically in Fig. 1b. Each center has used their own in-house treatment planning software to reproduce treatment plans to deliver 6 Gy (geometry A) and 8 Gy (geometry B) within the SOBP with the widths of 6 and 4 cm, respectively. More precisely, the widths and the locations of the SOBPs were defined through the depths of the 90 % dose levels (R90) (R90_distal_ = 109.5 mm, ΔR90 = 62.3 mm for 6 Gy plan and R90_distal_ = 125.6 mm, ΔR90 = 43.3 mm for 8 Gy plan), and the lateral field size was kept as 60 mm x 80 mm for both configurations. For every plan geometry, the slots for the slides were chosen to cover different positions in depth, namely four positions located in various regions of the SOBP, as well as three positions within the entrance channel region and one in the distal fall-off.

### Center-specific depth-RBE distributions

The data from the proton and X-ray experiments was used to calculate the values of proton RBE at different depths of each experimental field for every center. We have used the definition of the RBE as the ratio of photon and proton doses resulting in the same cell survival, and the approach is schematically explained in Fig. S1. For every center, we have first taken the average survival measured at a given depth of the proton field, and have extracted the corresponding isoeffective X-ray dose from the LQ fit of respective photon survival data. Next, the proton dose for a given depth was extracted from the corresponding planned depth-dose distribution. Finally, the center-specific RBE at a given depth was calculated as the ratio of the isoeffective X-ray and proton doses, with its error estimated from uncertainty propagation accounting for 5 % assumed uncertainty in the proton dose and fit errors of *α* and *β* parameters in the X-ray dose estimation.

## Results

### Variability in planned proton dose distributions across centers

**Fig. 2:**
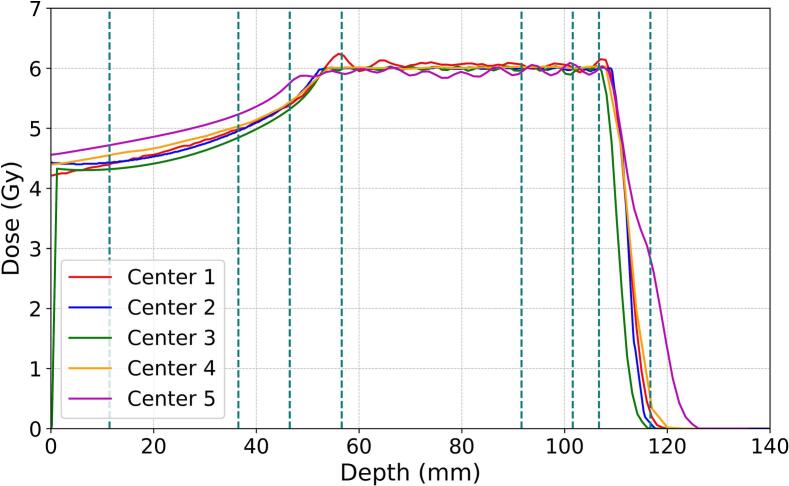

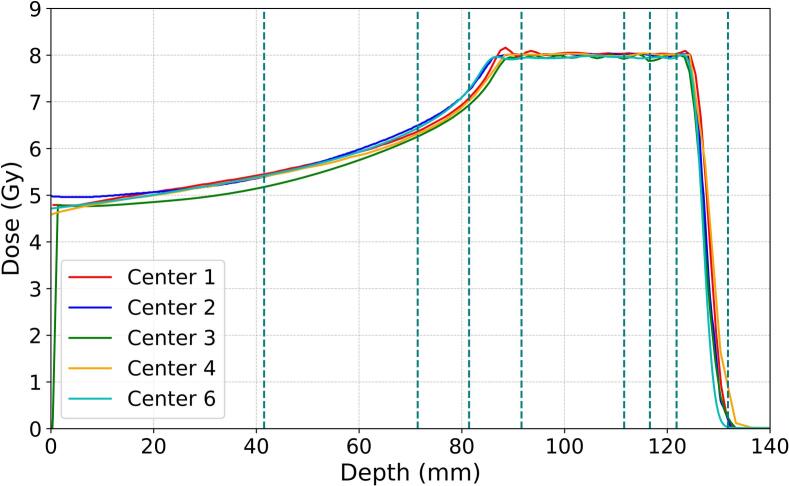
Planned depth-dose distributions for geometry A (6 Gy) and geometry B (8 Gy) with dashed lines indicating the positions of cell monolayers.

The planned dose distributions from every center are presented in Fig. 2A and 2B for the 6 Gy and 8 Gy plans, respectively.

There are larger discrepancies between the dose distributions for the 6 Gy plan compared to the 8 Gy plan, primarily in the entrance channel area. In particular, 6 Gy plan from Center 5 showed a significant dose increase of 5 − 7 % along the entrance channel, a more extended dose fall-off as well as a slightly wider SOBP compared to the other centers.

### Large spread in α/β values of the X-ray reference data

Prior to analyzing the survival measurements, plating efficiency (PE) was assessed for unirradiated control cells at each center. The PE values ranged from approximately 46 % to 65 % for both proton and X-ray setups. The absolute differences between proton and photon PE values at individual centers ranged from 5 % to 13 %. Despite this variability, the mean PE values across centers were comparable for protons and X-ray experiments (56 % and 58 %, respectively). These findings provide a reference for the interpretation of the survival measurements presented below.

**Fig. 3:**
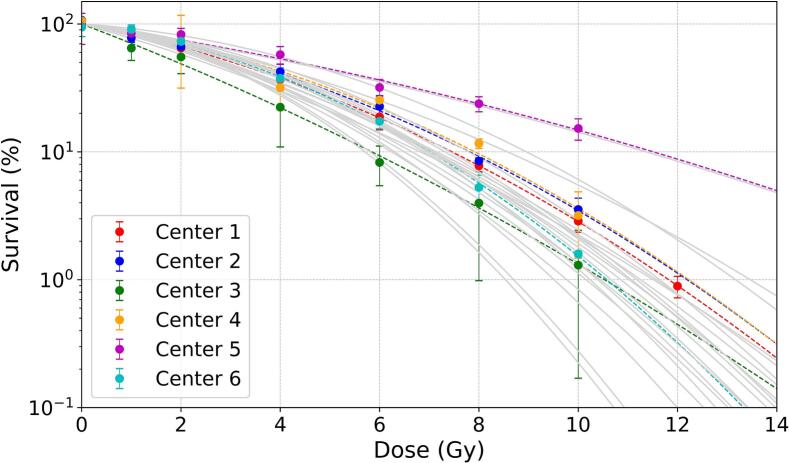
Survival fraction of V79-4 cells following X-ray irradiations. Dashed lines represent the LQ fit and the bars illustrate standard deviation (SD). Solid grey lines represent the X-ray response curves reported in the literature.

Fig. 3 shows X-ray survival values measured by every center and their fits with the LQ model. Despite the seemingly close measured survival values for the majority of the centers, the fit of the data demonstrated a large spread in the parameters, summarized in Supplementary Table 2. The steepest ’shoulder’ shape is observed by the Center 6 (α/β = 3.89 Gy), while the closest to a linear-shaped curve belongs to the Center 5 (α/β = 23 Gy). Grey lines correspond to the previously reported V79 X-ray response data, extracted from the PIDE database [13].

### Significant variations in proton survival measured along the beam path

**Fig. 4:**
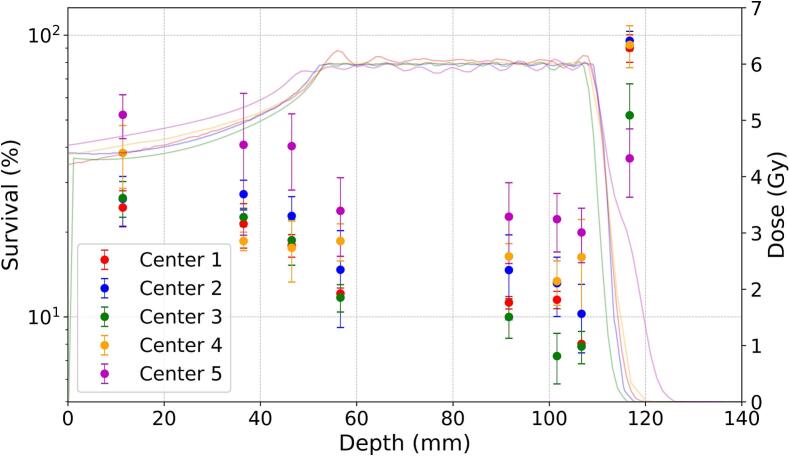

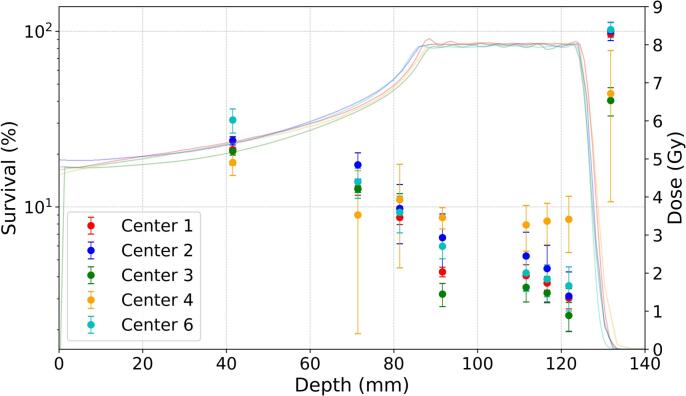
V79-4 cell survival distributions for 6 Gy (A) or 8 Gy (B) proton SOBP irradiations at eight different depths. The points represent the average measurements of each institute while the whiskers represent the standard deviations. Lines represent the respective depth-dose distributions for each center.

Fig. 4A and B represent the V79 survival distributions along the beam path from the 6 Gy and 8 Gy proton plans, respectively. For the Geometry A, the lowest survival in the SOBP region was measured by the Center 3. Center 5 measured significantly higher survival values compared to other participants despite the higher planned dose in the entrance channel. However, these measurements are in line with the results of the X-ray experiment, where the cells in Center 5 demonstrated reduced radiosensitivity. Nevertheless, most of the institutes seemed to have comparable results that overlapped each other within one standard deviation. Another set of outlying values can be seen from Center 4 for Geometry B, where cell survival in SOBP region is considerably higher, and the size of standard deviation are also significantly larger than those provided by other institutes.

**Fig. 5:**
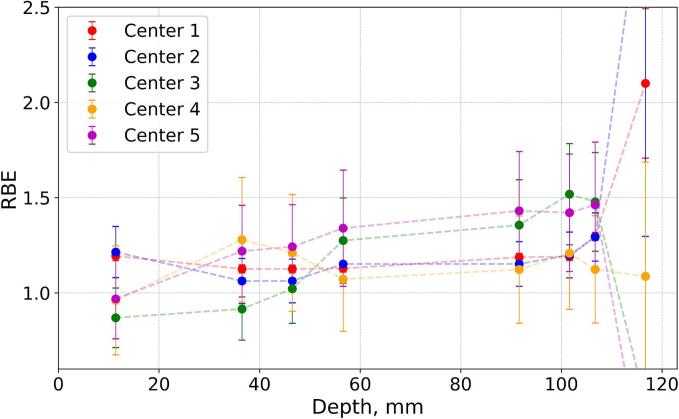

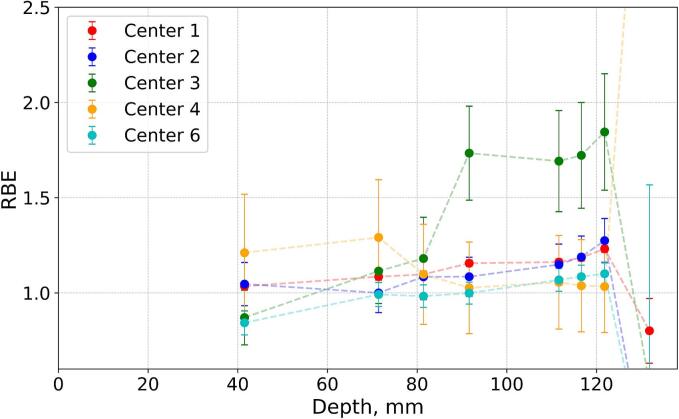
Proton RBE values estimated from the survival measurements for (A) 6 Gy and (B) 8 Gy plans, respectively. Error bars are calculated via the error propagation assuming the uncertainties in the proton and X-ray doses. Dashed lines are used for eye-guiding purpose.

While the overall picture depicted in Fig. 4, Fig. 5 may look comparable, a Kruskal-Wallis test (with α = 0.05) was also conducted to compare the individually measured survival values between the centers. For every depth, the survival measurements were found to be significantly different, as illustrated in Figs. S2 and S3 for 6 Gy and 8 Gy plans, respectively. Remarkably, the measurements performed at the distal edge region of both plans cannot provide any coherent conclusions, with the most probable reason attributed to a large variation in the delivered dose values on the short distance scale. The value dispersion is close to 70 − 80 % which makes the errors too large to recognize any trend.

### Depth distributions of proton RBE at different centers

Based on the acquired proton and X-ray data, we have calculated field-specific depth distributions of proton RBE for every center (Fig. 5). For both geometries (except for the depths corresponding to the SOBP fall-offs), the RBE values are in visually better agreement between the centers, as compared to the trends observed in the survival measurements, except for the Center 3 in the 8 Gy plan. There also might be an indication of an overall trend for the RBE values to slightly increase towards the distal edge of the SOBP (up to 1.5 and 1.3 for the 6 Gy and 8 Gy plans, respectively), in line with existing RBE-LET models. The two-way ANOVA (with α = 5 %) was performed for both cases, with two independent variables: depth and centers (Supplementary Table 3). The data points corresponding to the distal edge were not considered. The test revealed that differences between centers are statistically significant in both plans (similar to the results of Kruskal-Wallis test for survival). RBE variation along depth was significant and so was the link between depth and centers for the 8 Gy case, but not the 6 Gy case.

## Discussion

Our study aimed at comparing the in vitro survival measurements following proton irradiations at European facilities in harmonized experimental conditions, which was achieved by utilizing a cell phantom along with an elaborate study protocol. Despite the attempt to harmonize most of the experimental parameters, typically reported for this kind of proton irradiation, data showed significant heterogeneity. Namely, all survival measurements at every depth in the proton field showed significantly different results across centers, compromising the foundation of an equivalent dose response relationship within proton in vitro experiments when conducted across multiple institutions. For the 6 Gy plan, the coefficients of variation (CV), estimated for each measured depth as the ratios of standard deviations to the mean survival values for five centers, lay between 31–43 % across different depths, with the highest variation observed in the most distant point of the SOBP. The same was the case for the 8 Gy plan, however, there the CV value has reached 60 %. Generally, for this plan, there was more variation observed in the SOBP than in the plateau region (35–60 % compared to 10–23 %, respectively).

While the calculated RBE values might look comparable and follow the same trend, unfortunately, they are not statistically similar either. Therefore, our findings can be interpreted as the actual biological variations we have to deal with when most of the commonly reported parameters are aligned, which is important for interpretation of in vitro data and comparison between different studies (not just between facilities).

The heterogeneity highlights a well-known issue within radiobiological in vitro studies: the importance of considering the biochemical and biophysical context in which they were generated. A comparison of in vitro measurements, in this case, cell survival fractions, across different institutions is challenging, because this type of variability is usually not accounted for. The different survival values obtained in our study, with almost identical protocols and mandatory use of representative controls, underlines the problem of reproducibility in in vitro studies. While some of the observed differences might be attributed to biological variation, it seems however unlikely that cell lines of identical strand, bought from the same vendor, at the same time, could have explained such a remarkable difference in radiosensitivity. It seems more likely that minor differences in cell handling procedure, not specified in the harmonized protocol, have a more significant impact on results. Besides, despite the attempts to harmonize most of the experimental settings across centers, there was some room for variation left.

Firstly, the harmonization of the experimental procedures in this study was focused mainly on the biological setups. With regards to the physics setup, as it is commonly reported in the similar studies, we have fixed the depth and the width of the proton SOBP, but eventually observed variations in the dose distributions in the entrance channel or in the distal fall-off, which in turn would affect the measured survival. Moreover, the differences in the planned dose distributions, coupled with those in the beam delivery method and beam characteristics can lead to minor differences in LET distributions, consequently affecting the measured RBE. Although a detailed assessment of LET variations was beyond the scope of this study – requiring significantly larger experimental efforts beyond standard dosimetric quality assurance at each center – previous independent microdosimetric studies have shown that SOBPs generated by protons of similar energies at different facilities exhibit highly comparable mean lineal energy distributions [23,24]. Additionally, a study comparing actively scanned and passively scattered proton beams found no significant differences in clonogenic survival between the two delivery methods [25]. These findings suggest that minor variations in beam energies and irradiation modes are unlikely to be the primary source of the observed uncertainties in our study. Nevertheless, it is increasingly recommended to report an extended set of physical parameters when publishing particle irradiation data [26]. A detailed characterization and reporting of beam quality, ideally including also microdosimetric spectrum [27,28], derived from measurements or Monte Carlo simulations, would facilitate high quality biological data and will improve the quality of the intercomparisons.

Furthermore, some difficulties in the X-ray response may arise from the difference in the X-ray sources used [29], consequently affecting the calculated RBE values. However, based on the data in Fig. 3 we cannot conclude that one type of sources results in overall higher or lower reference survival values. The majority of the measured X-ray response curves align with the previously reported data for V79 cells. Furthermore, one of the centers has both X-rays facilities available (200 kV and 6 MV) and performed two types of measurements, finding no statistically significant differences between the two resulting datasets (Fig. S4).

In case of the manual cell- or colony counting, intra- and inter-observer variations between different centers can also play a role, with the second most likely having a higher impact, since the scientists within the same center are normally instructed in a similar way. For example, Fig. S5 shows the proton cell survival based on the indepented colony counts by two persons in Center 1, suggesting no significant differences between the two datasets. Nevertheless, standard counting procedures using well-calibrated automated tools would be highly desired for this type of studies.

Another factor that could contribute to the uncertainties can be the starting time of the sample post-processing due to the differences in radiation protection rules between the participating centers, setting the requirements for the waiting time after the irradiation (up to two hours at certain facilities), which might also affect the measured cell survival [30].

Moreover, in studies of this nature, a high number of experimental repetitions is crucial for improving the goodness of fit of X-ray survival curves and reducing error bars in the proton data. However, given the total number of data points required for both protons and X-rays, ensuring a high number of repetitions presents a significant challenge, since large numbers of samples are often difficult to accommodate due to limited laboratory capacities. Additionally, practical constraints such as restricted beamtime hours for protons or clinical linac X-rays further complicate the feasibility of multiple experimental repetitions.

All in all, our results emphasize the need for increasing the level of detail in describing the experimental conditions when publishing this kind of results. Researchers must be aware of the impact of the biological setup as well as the potential differences in beam characteristics on the measured data. These findings do not mean that the currently published in vitro data is not reliable, making the current RBE models unacceptable for clinical translation. Instead, because the inter-facility variability is very high, we rather suggest that every facility should measure biological response in vitro locally, especially is the facility is willing to implement a radiobiological model in the clinical treatment planning.

Conducting this multi-center intercomparison experiment required extensive coordination between biologists and physicists at multiple institutions, as well as careful standardization of experimental protocols. While this posed logistical challenges, such as increased complexity in scheduling, communication, and data exchange, it ultimately ensured a high level of methodological rigor. The successful execution of the study across several independent facilities strengthens the robustness of our findings by accounting for inter-institutional variations that single-center studies cannot capture, providing a realistic representation of the biological variability encountered in proton therapy research.

Despite these strengths, multi-center studies are often not prioritized by institutions, and unforeseen difficulties – such as beam time cancellations, dosimetry issues, biological variability, or personnel rotation – can cause delays or even experiment cancellations. Our study, initiated over four years ago, yielded sufficient data from only six participating centers, despite a larger number of institutions with suitable infrastructure. Future multi-center radiobiological studies might benefit from a traveling researcher format, where a single scientist conducts experiments across all sites. This structure could minimize heterogeneity from multiple cell handlers, reduce inter-observer variations (if these are not the study focus), and improve communication and logistical coordination.

## Conclusion

Despite the standardization efforts, significant variability in measured cell survival and RBE was observed across different European centers. This heterogeneity underscores the challenges in achieving reproducible in vitro radiobiological studies, even when utilizing harmonized experimental protocols. The observed differences highlight the potential impact of minor variations in experimental handling and local irradiation setups. The results stress the importance of detailed reporting in experimental procedures.

## Author contribution

MD and KK concevied the experiment; OS and NH designed the experimental protocol; OS collected and analyzed the data; MS perfomed the statistical analysis on the RBE data; ATF, OS and MS wrote the first draft; all authors participated in the experiments and edited the manuscript.

## Funding

This project has received funding from the European Union’s Horizon 2020 research and innovation programme under grant agreement No [730983] and grant from the Swedish Cancer Society (22 2365 PJ, 21 0371 FE, 24 3787 PJ) and the Swedish Childhood Cancer Fund (FT2023-0023, PR2023-0111). The publication is funded by the Open Access Publishing Fund of GSI Helmholtzzentrum fuer Schwerionenforschung.

## Declaration of competing interest

The authors declare that they have no known competing financial interests or personal relationships that could have appeared to influence the work reported in this paper.

## Appendix A Supplementary material

Supplementary Fig. 1 Schematic explanation the approach to calculate center-specific RBE distributions for each of the proton field configurations. The center-specific RBE at a given depth was calculated as the ratio of the isoeffective X-ray and proton doses. For every center, we have taken the survival measured at a given depth of the proton field, and the respective isoeffective X-ray dose was extracted from the LQ fit of X-ray survival data. The proton dose at a given depth was taken from the planned depth-dose distribution.

Supplementary Fig. 2 Individual survival values measured by each participating center at different depths for a 6 Gy SOBP proton field. Depths of 11.4 46.5 mm correspond to the entrance channel, 56.6 106.7 mm – to the SOBP, 116.7 mm – to the distal fall-off. Stars represent the results of a Kruskal-Wallis statistical analysis.

Supplementary Fig. 3 Individual survival values measured by each participating center at different depths for an 8 Gy SOBP proton field. Depths of 41.5 81.4 mm correspond to the entrance channel, 91.6 121.8 mm – to the SOBP, 131.8 mm – to the distal fall-off. Stars represent the results of a Kruskal-Wallis statistical analysis.

Supplementary Fig. 4 Survival fraction of V79-4 cells following X-ray irradiations measured at Center 4 with either 200 kV machine or 6 MV LINAC.

Supplementary Fig. 5 Survival fraction of V79-4 cells following proton irradiations measured at Center 1, resulting from independent cell counts performed by two different operators.

## References

[1] Grau C., Durante M., Georg D., Langendijk J.A., Weber D.C. (2020). Particle therapy in Europe. Mol Oncol.

[2] Particle Therapy Co-Operative Group. Particle therapy facilities in clinical operation. Accessed August 10, 2023. https://www.ptcog.site/index.php/facilities-in-operation-public.

[3] ICRU 78. Prescribing, Recording, and Reporting Proton-Beam Therapy: Contents.; 2007.

[4] Paganetti H., Blakely E., Carabe-Fernandez A. (2019). Report of the AAPM TG-256 on the relative biological effectiveness of proton beams in radiation therapy. Med Phys.

[5] Sørensen B.S., Pawelke J., Bauer J. (2021). Does the uncertainty in relative biological effectiveness affect patient treatment in proton therapy?. Radiother Oncol.

[6] Scholz M, Kellerer a. M, Kraft-Weyrather W, Kraft G. Computation of cell survival in heavy ion beams for therapy. *Radiat Environ Biophys*. 1997;36(1):59-66. doi:10.1007/s004110050055. 10.1007/s004110050055 9128899

[7] Hawkins R.B. (1998). A microdosimetric-kinetic theory of the dependence of the RBE for cell death on LET. Med Phys.

[8] Wilkens J.J., Oelfke U. (2004). A phenomenological model for the relative biological effectiveness in therapeutic proton beams. Phys Med Biol.

[9] Wedenberg M., Lind B.K., Hårdemark B. (2013). A model for the relative biological effectiveness of protons: the tissue specific parameter *α* / *β* of photons is a predictor for the sensitivity to LET changes. Acta Oncol (madr).

[10] McNamara A.L., Schuemann J., Paganetti H. (2015). A phenomenological relative biological effectiveness (RBE) model for proton therapy based on all published *in vitro* cell survival data. Phys Med Biol.

[11] Henthorn N.T., Gardner L.L., Aitkenhead A.H. (2023). Proposing a clinical model for RBE based on proton track-end counts. Int J Radiation Oncol *Bio*Phys.

[12] Carabe A., Moteabbed M., Depauw N., Schuemann J., Paganetti H. (2012). Range uncertainty in proton therapy due to variable biological effectiveness. Phys Med Biol.

[13] Friedrich T., Scholz U., ElsaSser T., Durante M., Scholz M. (2013). Systematic analysis of RBE and related quantities using a database of cell survival experiments with ion beam irradiation. J Radiat Res.

[14] Tommasino F., Durante M. (2015). Proton radiobiology. Cancers (Basel).

[15] Paganetti H., Niemierko A., Ancukiewicz M. (2002). Relative biological effectiveness (RBE) values for proton beam therapy. Int J Radiation Oncol *Bio*Phys.

[16] Lühr A., von Neubeck C., Pawelke J. (2018). “Radiobiology of Proton Therapy”: results of an international expert workshop. Radiother Oncol.

[17] Henthorn N.T., Sokol O., Durante M. (2020). Mapping the future of particle radiobiology in Europe: the INSPIRE project. Front Phys.

[18] Elsässer T., Weyrather W.K., Friedrich T. (2010). Quantification of the relative biological effectiveness for ion beam radiotherapy: direct experimental comparison of proton and carbon ion beams and a novel approach for treatment planning. Int J Radiat Oncol Biol Phys.

[19] Krämer M., Jäkel O., Haberer T., Schardt D., Weber U. (2000). Treatment planning for heavy-ion radiotherapy: physical beam model and dose optimization. Phys Med Biol.

[20] Krämer M., Scholz M. (2006). Rapid calculation of biological effects in ion radiotherapy. Phys Med Biol.

[21] Sørensen B.S., Vestergaard A., Overgaard J., Præstegaard L.H. (2011). Dependence of cell survival on instantaneous dose rate of a linear accelerator. Radiother Oncol.

[22] Fowler J.F. (1989). The linear-quadratic formula and progress in fractionated radiotherapy. Br J Radiol.

[23] Conte V., Bianchi A., Selva A. (2019). Microdosimetry at the CATANA 62 MeV proton beam with a sealed miniaturized TEPC. Phys Med.

[24] Rollet S., Colautti P., Grosswendt B. (2011). Microdosimetric assessment of the radiation quality of a therapeutic proton beam: comparison between numerical simulation and experimental measurements. Radiat Prot Dosim.

[25] Michaelidesová A., Vachelová J., Klementová J. (2020). In vitro comparison of passive and active clinical proton beams. Int J Mol Sci.

[26] Durante M., Paganetti H., Pompos A., Kry S.F., Wu X., Grosshans D.R. (2019). Report of a National Cancer Institute special panel: characterization of the physical parameters of particle beams for biological research. Med Phys.

[27] Magrin G., Palmans H., Stock M., Georg D. (2023). State-of-the-art and potential of experimental microdosimetry in ion-beam therapy. Radiother Oncol.

[28] Lühr A., Wagenaar D., Eekers D.B.P. (2025). Recommendations for reporting and evaluating proton therapy beyond dose and constant relative biological effectiveness. Phys Imaging Radiat Oncol.

[29] Paget V., Ben Kacem M., Dos Santos M. (2019). Multiparametric radiobiological assays show that variation of X-ray energy strongly impacts relative biological effectiveness: comparison between 220 kV and 4 MV. Sci Rep.

[30] Reddy N.M., Mayer P.J., Nori D., Lange C.S. (1995). Chinese hamster V79 cells harbor potentially lethal damage which is neither fixed nor repaired for long times after attaining maximal survival under growth conditions. Radiat Res.

