# Supplementary figures and images for: In vitro measurement of proton RBE: A multi-centric comparison using a harmonized setup
Source: Clin Transl Radiat Oncol. 2025 May 11;53:100978. doi: 10.1016/j.ctro.2025.100978 (PMC12141100; doi:10.1016/j.ctro.2025.100978)

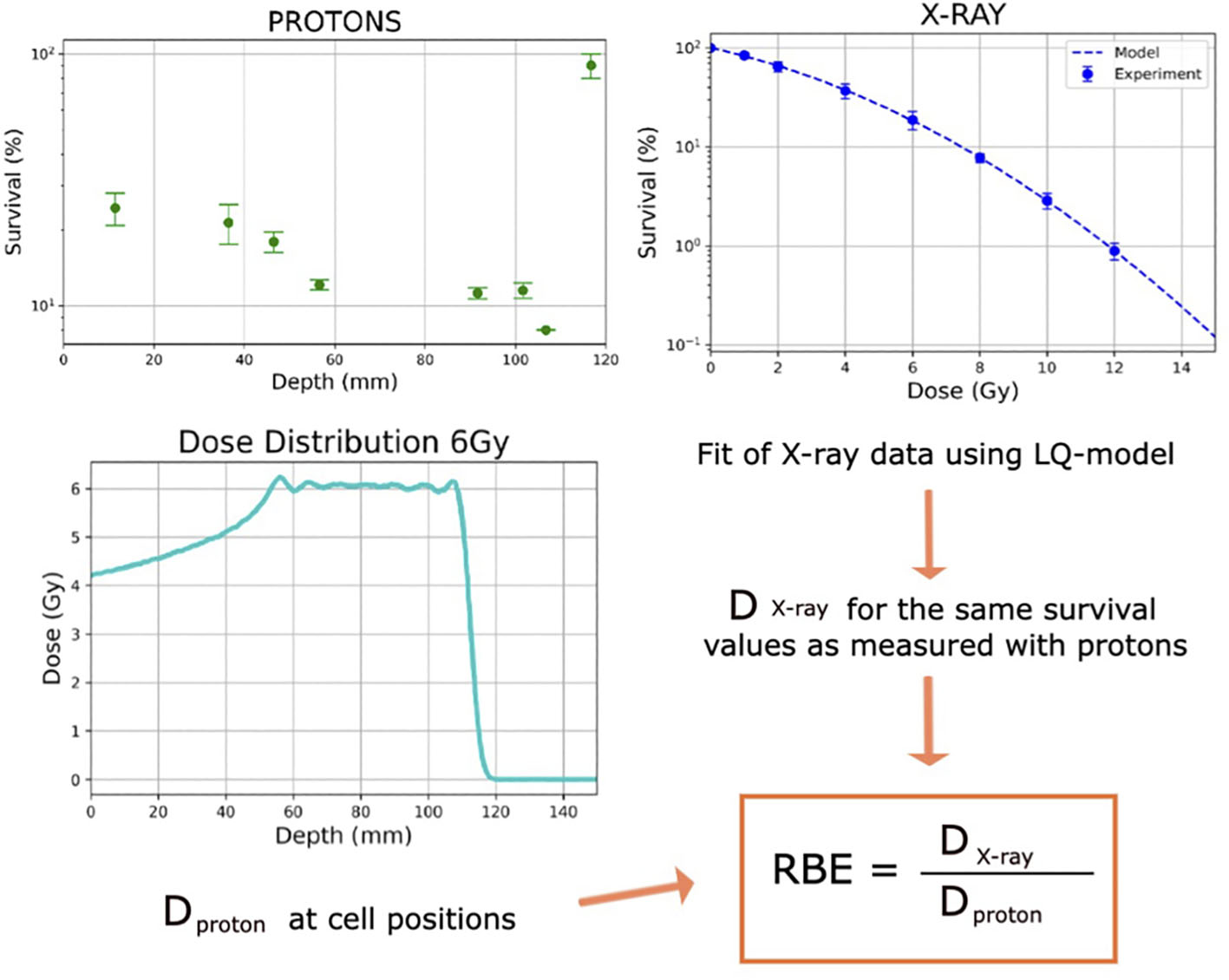

Supplement: Supplementary Fig. 1 — Schematic explanation the approach to calculate center-specific RBE distributions for each of the proton field configurations. The center-specific RBE at a given depth was calculated as the ratio of the isoeffective X-ray and proton doses. For every center, we have taken the survival measured at a given depth of the proton field, and the respective isoeffective X-ray dose was extracted from the LQ fit of X-ray survival data. The proton dose at a given depth was taken from the planned depth-dose distribution. [file mmc4.jpg]

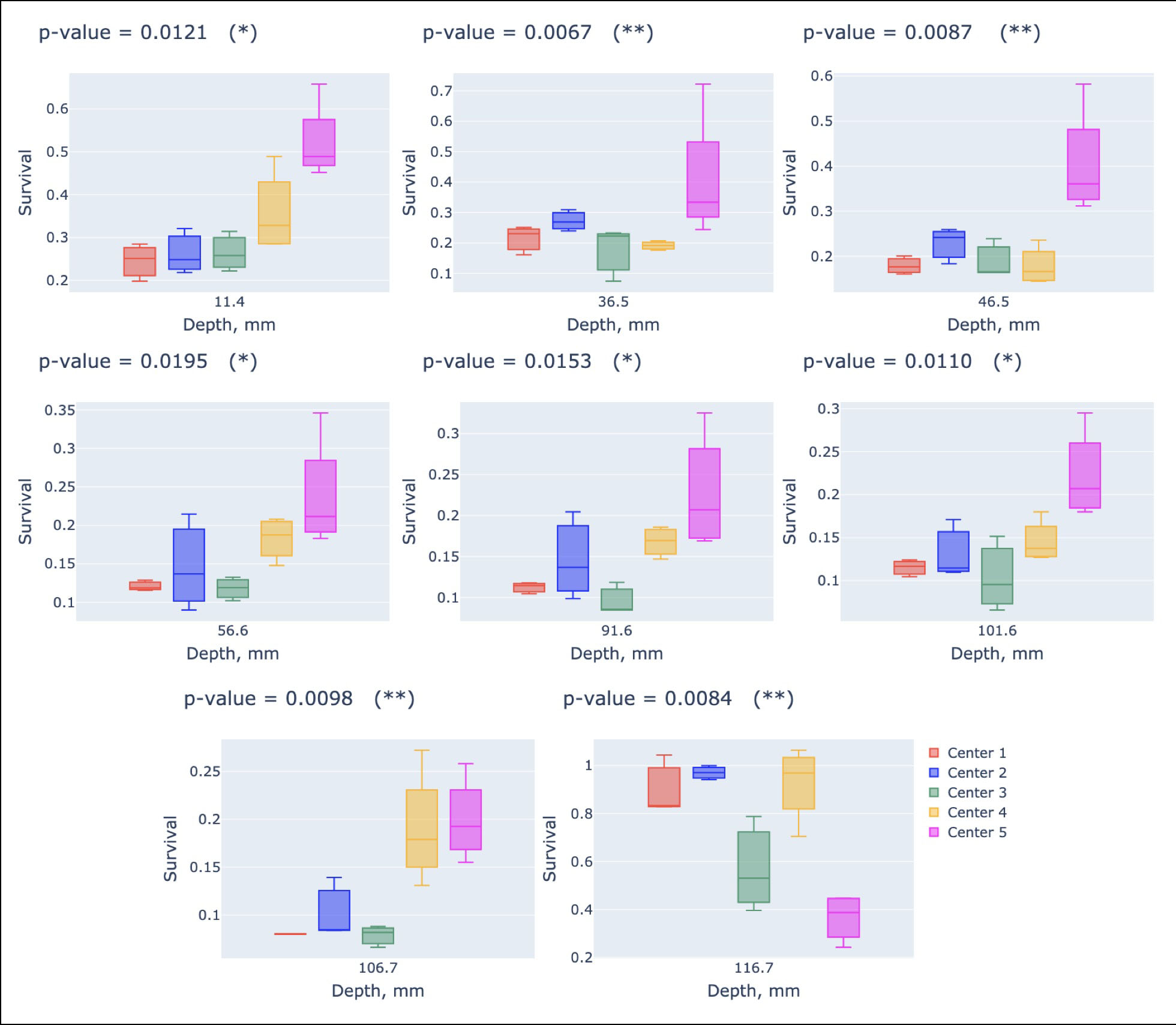

Supplement: Supplementary Fig. 2 — Individual survival values measured by each participating center at different depths for a 6 Gy SOBP proton field. Depths of 11.4 46.5 mm correspond to the entrance channel, 56.6 106.7 mm – to the SOBP, 116.7 mm – to the distal fall-off. Stars represent the results of a Kruskal-Wallis statistical analysis. [file mmc5.jpg]

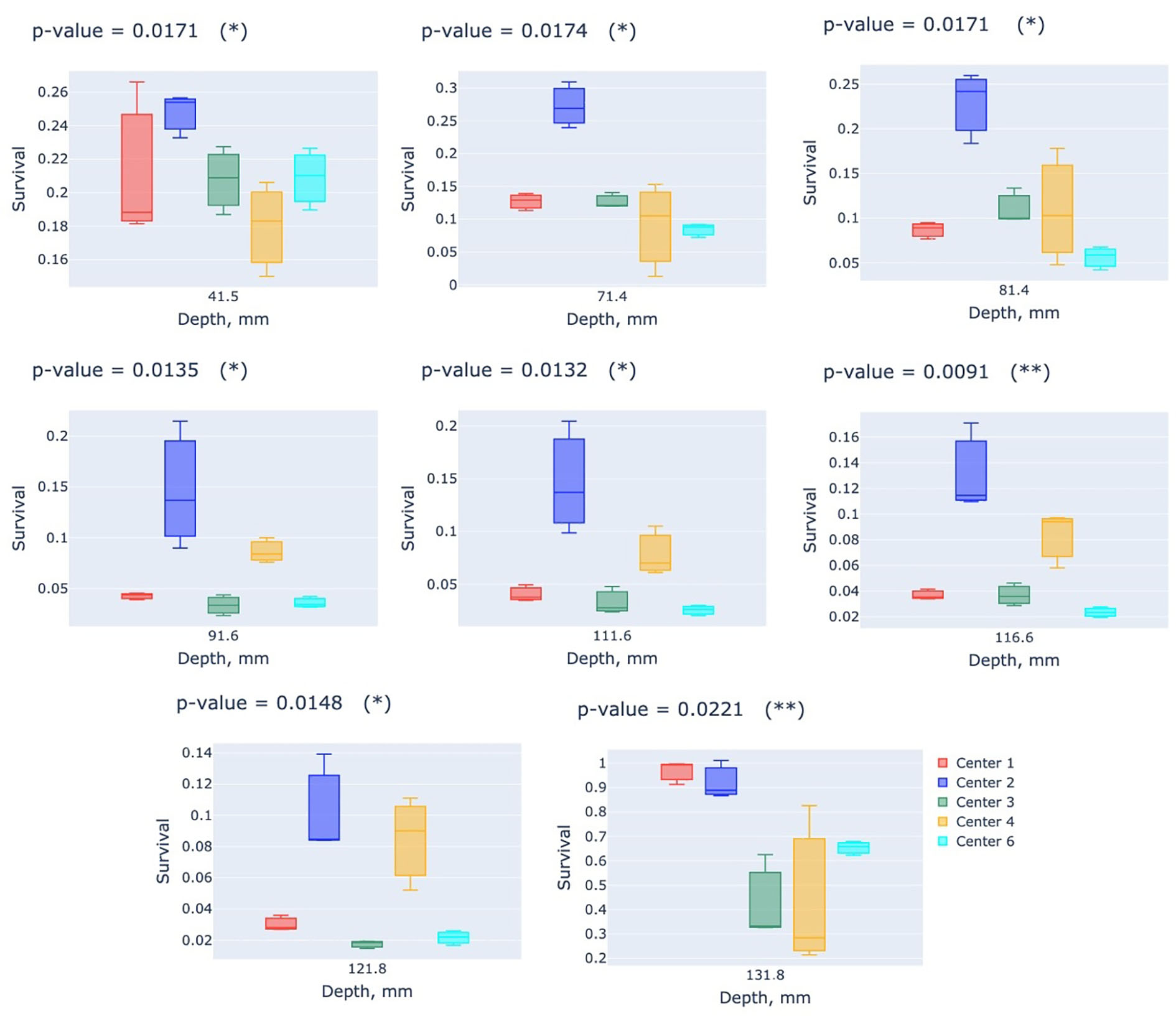

Supplement: Supplementary Fig. 3 — Individual survival values measured by each participating center at different depths for an 8 Gy SOBP proton field. Depths of 41.5 81.4 mm correspond to the entrance channel, 91.6 121.8 mm – to the SOBP, 131.8 mm – to the distal fall-off. Stars represent the results of a Kruskal-Wallis statistical analysis. [file mmc6.jpg]

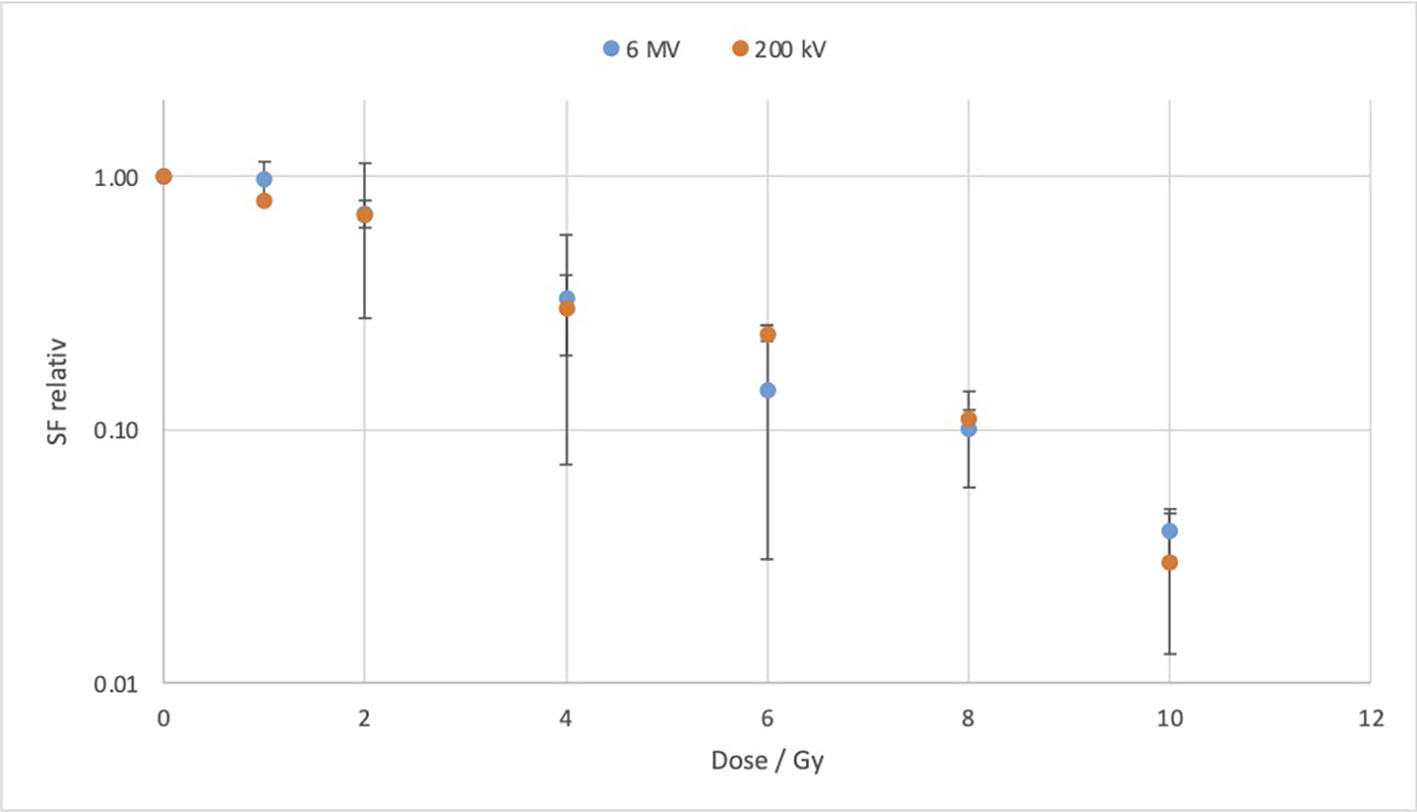

Supplement: Supplementary Fig. 4 — Survival fraction of V79-4 cells following X-ray irradiations measured at Center 4 with either 200 kV machine or 6 MV LINAC. [file mmc7.jpg]

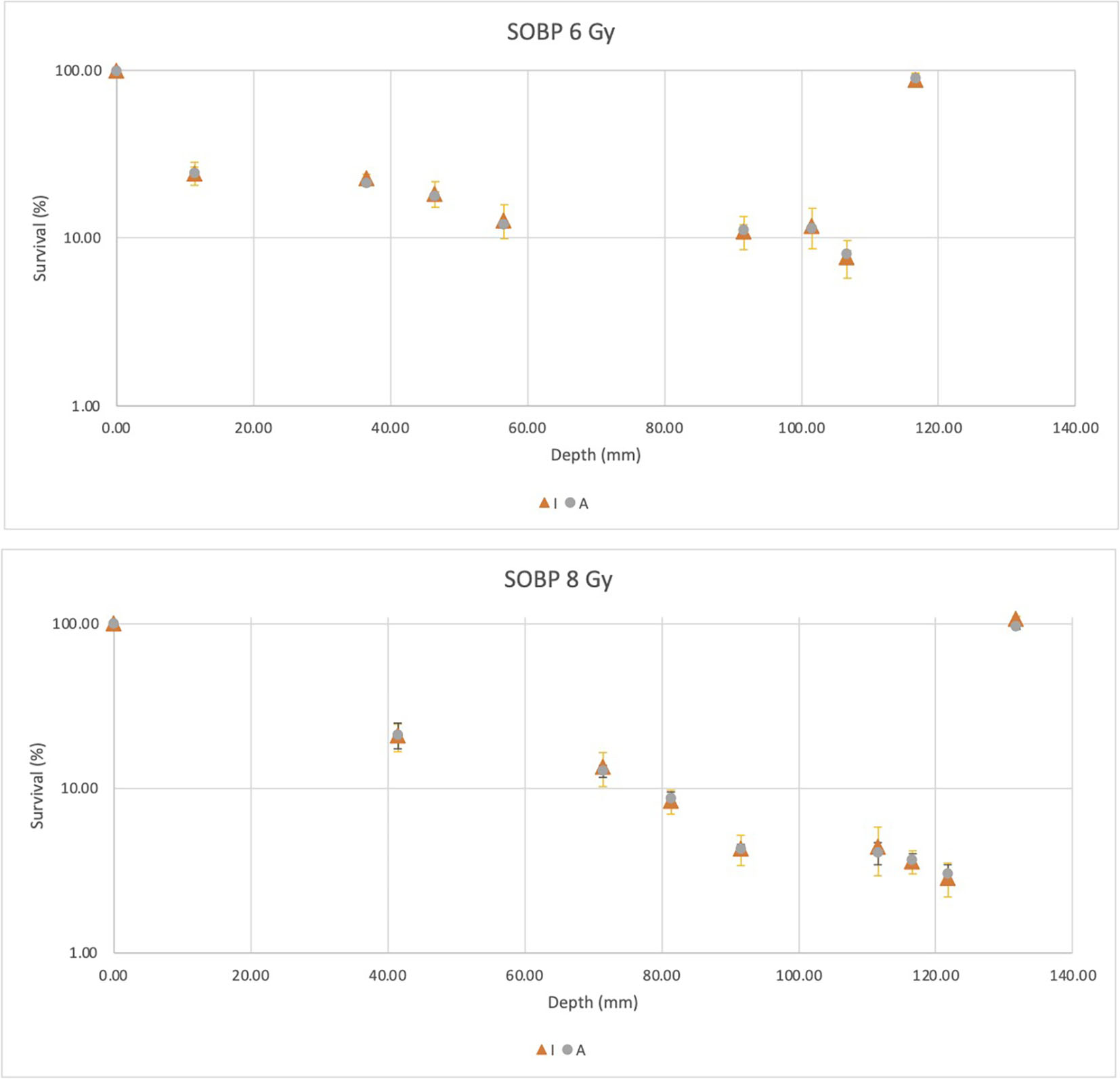

Supplement: Supplementary Fig. 5 — Survival fraction of V79-4 cells following proton irradiations measured at Center 1, resulting from independent cell counts performed by two different operators. [file mmc8.jpg]
