# Supplementary material for: In vitro measurement of proton RBE: A multi-centric comparison using a harmonized setup
Source: Clin Transl Radiat Oncol. 2025 May 11;53:100978. doi: 10.1016/j.ctro.2025.100978 (PMC12141100; doi:10.1016/j.ctro.2025.100978)
Supplement: Supplementary Data 1 [file mmc1.docx]

| **Center** | **X-rays** | **Protons** |
| --- | --- | --- |
| 1 | 4 | 3 |
| 2 | 3 | 3 |
| 3 | 3 | 3 |
| 4 | 3 | 3 |
| 5 | 3 | 4 |
| 6 | 2 | 3 |

***Supplementary Table 1****. Number of independent repetitions of the X-rays and each of the proton experiments performed at each participating center.*
