# Supplementary material for: In vitro measurement of proton RBE: A multi-centric comparison using a harmonized setup
Source: Clin Transl Radiat Oncol. 2025 May 11;53:100978. doi: 10.1016/j.ctro.2025.100978 (PMC12141100; doi:10.1016/j.ctro.2025.100978)
Supplement: Supplementary Data 2 [file mmc2.docx]

| **Center** | **α, Gy^−1^** | **∆α, Gy^−1^** | **β, Gy^−2^** | **∆β, Gy^−2^** |
| --- | --- | --- | --- | --- |
| 1 | -0.1709 | 0.0056 | -0.0185 | 0.0005 |
| 2 | -0.1470 | 0.0342 | -0.0189 | 0.0034 |
| 3 | -0.3403 | 0.0694 | -0.0092 | 0.0062 |
| 4 | -0.1328 | 0.0840 | -0.0199 | 0.0082 |
| 5 | -0.1334 | 0.0401 | -0.0058 | 0.0039 |
| 6 | -0.1172 | 0.0273 | -0.0301 | 0.0027 |

***Supplementary table 1****. V79-4 LQ parameters estimated from the X-ray survival curves at each participating center.*
