# Supplementary material for: In vitro measurement of proton RBE: A multi-centric comparison using a harmonized setup
Source: Clin Transl Radiat Oncol. 2025 May 11;53:100978. doi: 10.1016/j.ctro.2025.100978 (PMC12141100; doi:10.1016/j.ctro.2025.100978)
Supplement: Supplementary Data 3 [file mmc3.docx]

|  | **6 Gy plan** | **8 Gy plan** |
| --- | --- | --- |
| **Depths** | p=0.334512118 | p=2.953E-10 |
| **Centers** | p=0.000598852 | p=9.79524E-06 |
| **Interaction** | p=0.348913615 | p=5.77491E-05 |

***Supplementary table 2****. P-values of two way ANOVA for RBE values.*
